# Evaluation of an adaptive virtual laboratory environment using Western Blotting for diagnosis of disease

**DOI:** 10.1186/1472-6920-14-222

**Published:** 2014-10-20

**Authors:** Patsie Polly, Nadine Marcus, Danni Maguire, Zack Belinson, Gary M Velan

**Affiliations:** Department of Pathology, School of Medical Sciences, UNSW Medicine, UNSW Australia, Kensington, NSW 2052 Sydney Australia; Computer Science and Engineering, Faculty of Engineering, UNSW Australia, Kensington, NSW 2052 Sydney, Australia; Smart Sparrow, Surry Hills, NSW 2010 Sydney, Australia

**Keywords:** Pathology, Science education, Virtual laboratory, ELearning, Technical skills, Diagnostic skills, Western Blotting, Muscular dystrophy

## Abstract

**Background:**

Providing large numbers of undergraduate students in scientific disciplines with engaging, authentic laboratory experiences is important, but challenging. Virtual laboratories (vLABs) are a potential means to enable interactive learning experiences. A vLAB focusing on Western Blotting was developed and implemented in a 3rd year undergraduate Pathology course for science students to facilitate learning of technical molecular laboratory skills that are linked to development of diagnostic skills. Such skills are important for undergraduates in building a conceptual understanding of translation of laboratory techniques to changes in human biology due to disease.

**Methods:**

The Western Blotting vLAB was developed and deployed using the Adaptive eLearning Platform (AeLP) developed by Smart Sparrow (https://www.smartsparrow.com/). The vLAB was evaluated to assess students' perceptions of their laboratory skills relevant to the diagnosis of Muscular Dystrophy. A blended learning rotation model was applied in which wet laboratory and vLAB environments for Western Blotting were both delivered to three consecutive cohorts of 3rd year science undergraduates undertaking a Muscle Diseases practical class. Evaluation questionnaires were administered at the completion of the practical classes.

**Results:**

Students indicated in online questionnaires that the Western Blotting vLAB was at least equivalent to the real lab in their perceived development of concepts, laboratory skills and diagnosis of disease.

**Conclusions:**

vLABs have great potential for improving students’ development of diagnostic skills. Further studies are required to determine the impact of vLABs on student learning.

**Electronic supplementary material:**

The online version of this article (doi:10.1186/1472-6920-14-222) contains supplementary material, which is available to authorized users.

## Background

Providing large numbers of undergraduate students in scientific disciplines with engaging, authentic laboratory experiences is important to promote inquiry-based, conceptual learning [1], but challenging in terms of resourcing [2]. Constraints in a real lab (wet-lab) setting include limited and out-dated laboratory equipment, unavailable materials and difficulties in demonstrating techniques due to large class sizes resulting in student crowding [3]. These resourcing issues have implications for cost, health and safety as well as efficacy of teaching and learning. Such issues have affected the Muscle Diseases practical class within Musculoskeletal Diseases, an undergraduate Pathology course for 3rd year Science students at UNSW Australia. Student enrolments in the Musculoskeletal Diseases course have risen markedly over the past 5 years. This has made it logistically very difficult to teach technical and diagnostic laboratory elements and the use of protein analysis apparatus (Western Blotting) in that practical class. Such technical skills are linked to conceptual frameworks that underpin the diagnosis of muscular dystrophy. Furthermore, the use of Western Blotting apparatus in a real lab setting is time-consuming and cumbersome and tends to detract from the diagnostic aspect of the practical class [4].

Moreover, the focus of the labs is on acquiring technical and diagnostic skills, and the cognitive load imposed by learning to use real lab apparatus is possibly redundant to that goal [5]. This is not to say that the lab equipment is redundant overall, but creates extraneous load when trying to acquire diagnostic skills, as the focus of attention may be on mastering the technical aspects of the apparatus instead of understanding the protein expression patterns that underlie the diagnosis of muscular dystrophy. Hence, the concern was that learners may be distracted by focussing on equipment use in wet labs, rather than learning important concepts such as disease diagnosis in muscular dystrophy. Therefore, one potential benefit of virtual laboratories (vLABs) is that they allow for the separation of mastery of technical equipment from the acquisition of diagnostic skills. Another clear advantage of vLABs is the potential cost savings on consumables and laboratory demonstrators. In this context, vLABs are a potential means to enable cost-effective, safe and efficient, interactive learning experiences [6].

We opted to employ a vLAB format to overcome constraints with the real laboratory experience and to enable students to focus on difficult concepts more effectively, such as the threshold concept that underpins how changes in protein expression result in manifestation of disease at the gross anatomical level [4, 7]. Threshold concepts are those key concepts that transform thinking irreversibly but are often difficult to understand and acquire. Once acquired they may even seem simple and self-evident [8]. vLABs have already been trialed successfully within an Engineering context to improve the teaching of Mechanical Engineering threshold concepts [9]. Using well guided instructional techniques such as those described previously [10], vLABs also have the potential to reduce complexity [11] by providing increased opportunities for remediation and practice, as well as guided feedback [12]. They also allow for increased exposure and practice with threshold concepts. The Adaptive eLearning Platform (AeLP) [13, 14] can be used to develop vLABs that are adaptively sequenced to meet students’ individual learning needs, provide adaptive feedback to students on misconceptions, as well as tracking students’ interactions within the virtual environment. The key advantage of using the AeLP to create the Western Blotting vLAB was retention of pedagogical control over the laboratory environment and ‘in-the-moment’ feedback or adaptation to student interactions with the Western Blotting vLAB. Thus, there is emerging evidence that virtual laboratory environments are useful for learning threshold concepts. The AeLP is an innovative way of teaching such concepyts by providing adaptive feedback to students and addressing their needs as they progress through the vLAB.

We are not aware of any previous reports of evaluative data regarding the use of vLABs for Western Blotting, or similar molecular diagnostic techniques. To investigate whether such vLABs are acceptable and effective for learning, we developed a vLAB to demonstrate the process of analysing protein expression by muscle cells, which is a necessary step in the real-world diagnosis of muscular dystrophy and implemented it in a blended learning teaching environment. Hence, the focus of the Western Blotting vLAB was to teach principles of sodium-dodecyl-sulfate polyacrylamide gel electrophoresis (SDS-PAGE) and Western Blotting of dystrophin protein to analyse altered protein expression patterns in patients with disease.

Our anecdotal observations from earlier versions of the Muscle Diseases practical class that used the real lab alone, showed that students needed more guidance in the area of diagnosis of disease using laboratory techniques. This is important, as students need to understand that the quality of how laboratory techniques are performed can impact on the quality of the generated results and therefore outcomes of disease diagnosis. The aims of implementing the vLAB in this study were to: 1) improve students’ understanding of how differences in molecular signatures or altered protein expression patterns could be linked to microscopic or macroscopic changes in diseases such as muscular dystrophy; and 2) evaluate whether students perceived the vLAB as being more or less useful than the real lab in teaching concepts and skills related to Western Blotting.

## Methods

### Development of the Western Blotting vLAB

#### Development process

There were two key development considerations: 1. the broader lesson had to be developed in view of the Muscle Diseases Practical and overall PATH3207 Musculoskeletal Diseases course learning objectives and 2. the Western Blotting vLAB had to be designed to achieve integration with the lesson about the molecular basis of muscular dystrophy whilst keeping it generic enough to be reusable in slightly varying contexts, i.e. teaching of Western Blotting for the purposes of technical skills only. The Software Development Life Cycle (SDLC) method is described a series of phases providing a model for software development and management of the Western Blotting vLAB (Additional file 1).

#### Western Blotting vLAB design

The Western Blotting vLAB was designed as a flexible, integrated formative experience which combined introductory information, tasks with associated questions and various embedded media such as short videos to enhance student learning. Deployment flexibility entailed the ability to publish the practical lesson for students in-class. This was the original intent, but the capacity is also available to deploy the vLAB for preparation and/or revision purposes. A range of key criteria were utilised in the design of the Western Blotting vLAB, based on best practice in eLearning [12, 15, 16] (Additional file 2).

#### VLAB interface, environment and navigation

**Figure 1:**
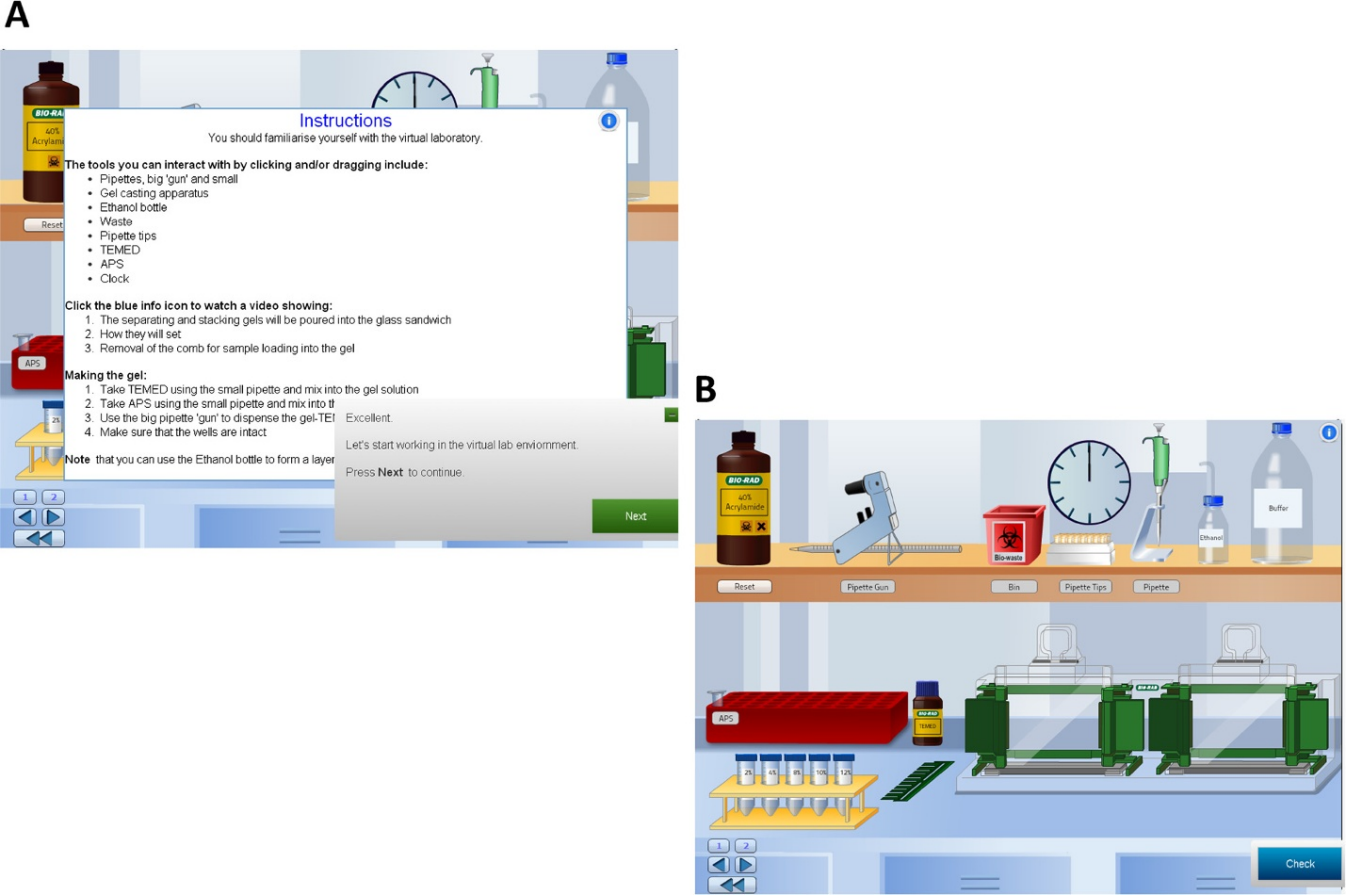
**Technical Instructions and Feedback.** Instructions and related feedback provided prior to students starting the vLAB are shown as inserted panels that overlay the vLAB scene **(A)**. The vLAB scene demonstrating reagents and SDS-PAGE gel units **(B)**.

**Figure 2:**
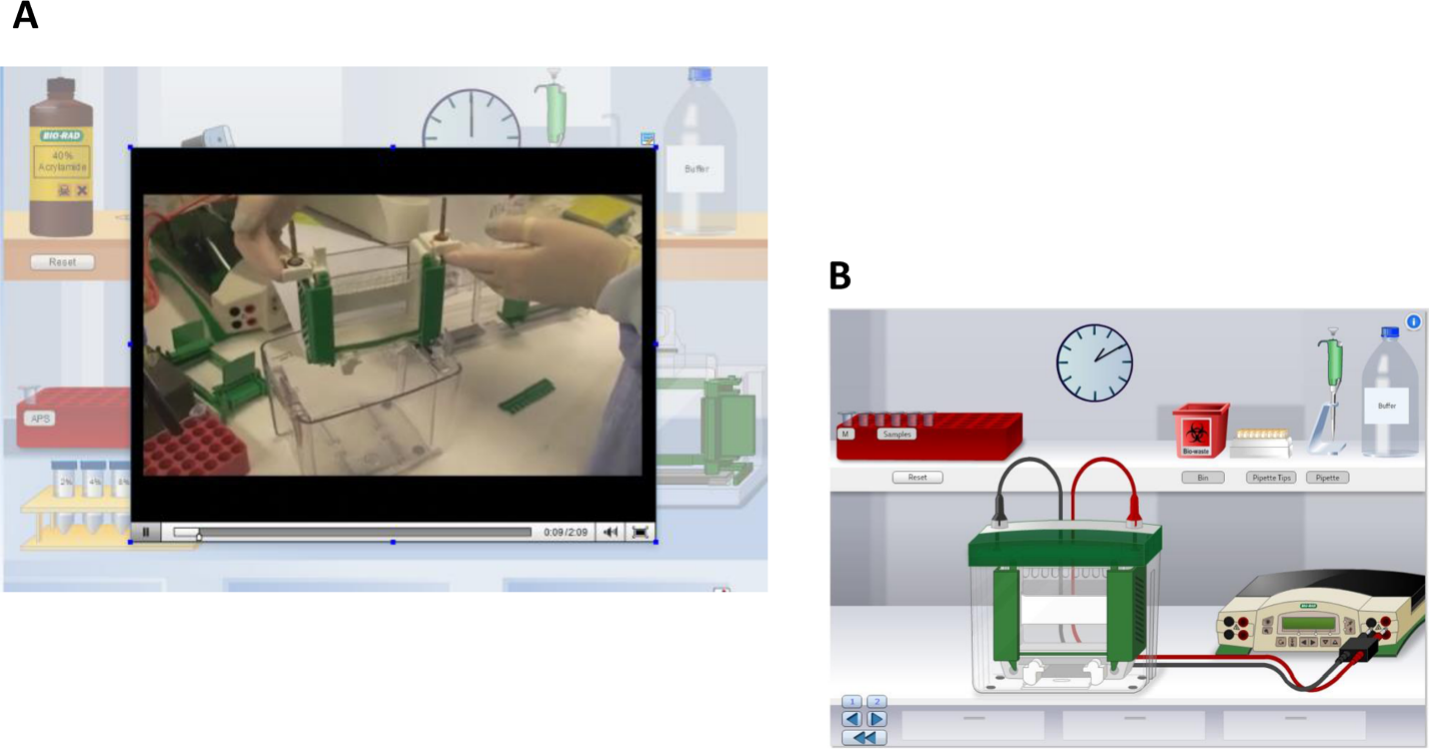
**Videos embedded in the vLAB.** Videos were embedded into certain areas of the vLAB to model technically challenging aspects of the Western Blotting experiment. Students were instructed to watch the video prior to starting the vLAB **(A)**. Modelling how the SDS-PAGE gel unit was assembled as part of the Western Blotting protocol is shown **(B)**.

**Figure 3:**
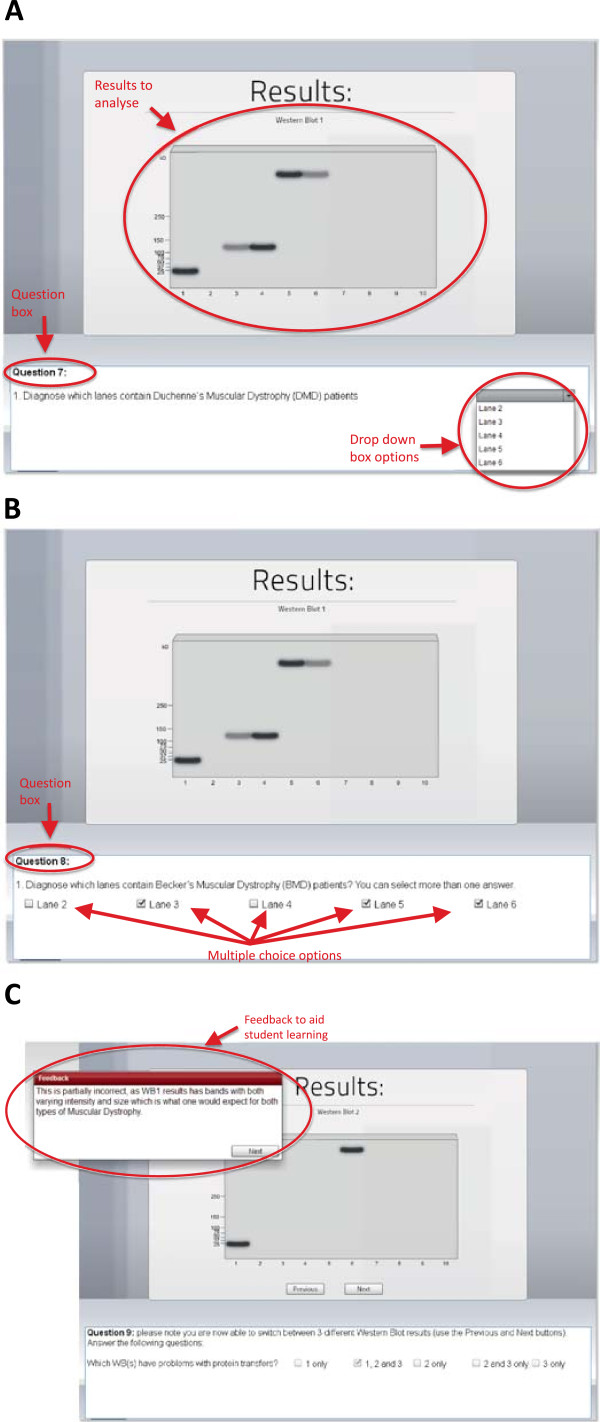
**Diagnosis of Muscular Dystrophy.** A series of multiple choice questions with drop-down menus were provided to the students with or without adaptive feedback to suit student choices is shown. A series of screens from the Western Blotting vLAB showing **(A-C)** ‘results’, **(A, B)** related questions and **(C)** tailored feedback is shown.

The Western Blotting vLAB interface was kept consistent and simple to avert excess cognitive load. The vLAB emulated the real lab environment with high fidelity in terms of the type of materials and reagents. We ensured that the appearance and technical function of laboratory apparatus and instruments mirrored the real world as shown in Figures 1, 2 and 3. This helped to eliminate any distractions from the core learning objectives of conceptual understanding of the molecular basis of muscular dystrophy as well as technical skills.

The introductory screens included learning objectives, instructions and videos (Figures 1 and 2) to quickly orientate the learner to the technical aspects of Western Blotting in the context of diagnosing muscular dystrophy. Continual functions included forward and back options, and a return to main menu button on each screen (Figure 1). Embedded video links facilitated demonstration of technically more challenging aspects of the lab so that learning bottlenecks could be overcome via modelling, thus enabling students to progress through the vLAB in a timely manner (Figure 2). These elements were inserted in order to support student use of the equipment in the vLAB and also had the effect of diversifying the mode of presentation to maintain student engagement. This design approach facilitated students’ control of their learning as well as providing additional information on a specific concept, e.g. explaining the significance of a particular technical parameter such as loading the samples correctly into the SDS-PAGE gel lanes. Moreover, videos or animations of tasks involving hand motor skills have been found to lead to increased learning when compared to static images [17, 18].

#### Feedback and interactive features

In the context of laboratory experiments, interactivity and feedback are core features in promoting learning. Immediate feedback is vital for the learning process of technical and diagnostic skills and for correcting mistakes and misconceptions [12, 15, 19, 20]. Throughout the vLAB, students need to interact with the laboratory environment and with the concepts related to interpreting the results. This is achieved by utilising a variety of question formats (e.g. multiple choice, drop-down lists) with immediate feedback following students’ submission of responses (Figure 3). Additionally, some screens did not permit progress unless the laboratory task was attempted and feedback received. In some instances, the screen was reset after feedback was received. This was intended to ensure that students engaged with the content at their own pace, rather than clicking through the laboratory environment without engaging with the technical and theoretical aspects of the laboratory. Self-paced, guided learning has been shown to improve learning [21]. The diagnosis of muscular dystrophy and related technical issues were addressed in the ‘*Results*’ section of the vLAB (Figure 3A, B, C). A series of multiple choice questions with drop-down menus shown in Figure 3A were provided to the students with adaptive feedback appropriate to students’ choices. As laboratory data showing various outcomes from each Western Blot were revealed on each successive screen, formative assessment questions and relevant feedback were presented regarding the diagnostic implications of protein expression patterns (Figure 3C).

### Deployment and evaluation

In 2011, we documented student user experience and engagement using questionnaires and in class observations of the vLAB use by students for the purposes of implementing improvements in subsequent deployments. Student feedback and technical observations from the first deployment in 2011 were taken into account before the second deployment in 2012. The third deployment in 2013 had no additional changes from 2012.

#### Student groups

**Figure 4:**
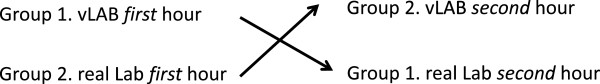
**Muscle Diseases Practical Class Structure for a Two hour Lesson.** Students were randomly divided into two groups, groups 1 and 2. The groups were crossed-over mid-way through the practical class to ensure that all students performed both types of Western Blotting laboratory.

The student cohorts undertaking the Musculoskeletal Diseases course in 2011 (n = 80), 2012 (n = 73) and 2013 (n = 59) were divided into two groups of approximately equal size for the Muscle Diseases practical classes. Group 1 was asked to attempt the vLAB first while group 2 completed the real lab. Halfway through the 2-hour class, groups 1 and 2 swapped lab environments (Figure 4). The same strategy was applied again in 2012 and 2013. We documented feedback on the logistics of running the vLAB in 2011 given by student users so that we could implement improvements in 2012 and again in 2013 which had the general effects of streamlining vLAB interactions and improving lesson flow.

#### Evaluation questionnaires

We designed a questionnaire addressing the technical and diagnostic skills covered by the vLAB. These questionnaires were provided electronically to all students upon completion of the practical class. The questionnaire provided in Additional file 3, used a combination of 3 point (eg. ‘Yes, Not sure, No’), 4 point (‘no it did not - > yes’) and 5-point Likert scale questions to enable students to report their perceived understanding, confidence and learning of technical and diagnostic skills in the vLAB and the real lab environments. This study was approved by the UNSW Human Research Ethics Committee (UNSW Ethics no. HC13004).

#### Statistical analysis

Mann–Whitney U tests were used to compare the distribution of ordinal questionnaire data between groups in each year. A *p* value of <0.05 was considered significant.

## Results

### Diagnostic skills

**Figure 5:**
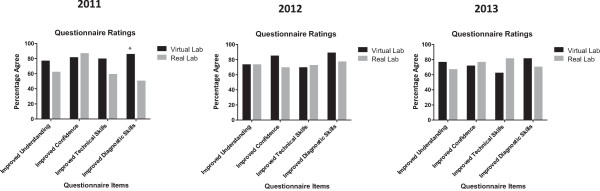
**Student Learning, Diagnostic Skills.** The distribution of Likert scale responses regarding learning of diagnostic skills for Western Blotting and protein expression analysis was significantly enhanced using the vLAB compared with the real lab in 2011 (*p = 0.006, Mann–Whitney U test). There were no other significant differences in distribution of Likert scale data between the vLAB and the wet lab for other items in all years.

Evaluation questionnaires revealed that students perceived that the most useful skills learned by using the vLAB included application of laboratory methods (i.e. Western Blotting) to the diagnosis of disease. These perceptions were significantly improved when compared with the real lab in 2011 (*p* = 0.006; Figure 5). Students also commented on the effects of the vLAB regarding interpretation of changes in protein expression due to disease:

*“It was good to apply the skills to why we were doing it and how it is used for actual diseases”; “Allowed me to see the band patterns of different muscle diseases”*; and *“Mistakes in the virtual lab can be addressed sooner than the wet lab. This saves time overall”.*

The vLAB also appeared to assist preparation prior attempting the real lab. This occured when students attempted the vLAB before the real lab in the Muscle Diseases practical:

*“The virtual lab broke down each section and explained it in more details so by the time you got to the wet lab you understood what was happening”; and “It helped me understand which steps I made silly mistakes in”.*

### Student understanding

The vLAB was equivalent to the real lab regarding students' perceptions of its utility in aiding their understanding of Western Blotting (Figure 5).

Students’ comments indicated that they valued learning the process of each step in the experiment:

*“It was fun to do it in a ‘hands on’ way so we understand the importance of each step”;* and *"The demonstrations explained the reason for each step and how to theoretically perform the method”.*

Students also commented on the flexibility and remedial value of the vLAB:

*“Could re-watch the videos which were clearly explained by the demonstrator”;*

*“Can make mistakes but still reach appropriate outcomes as we have the chance to re-do”;*

*“The virtual lab explained each step better with the videos”;*

*“Interactive learning experience, mimics exactly how a lab is. Great.” and “The opportunity to 'practice', without the pressure of wasting actual materials”.*

Some students commented on the effect of the vLAB on their understanding on the conceptual basis of Western Blotting, and how alterations in protein expression are linked to disease.

*“Learnt that gel casting protein transfer and loading can all influence the migration of a protein within developed gel and the expression of a given protein i.e. darkness of the band”; and*

*“Allowed me to see the band patterns of different muscle diseases”.*

Student comments on the effect of the vLAB on their perceived confidence in laboratory skills included:

*“It was better than the real life version”; and “Gave more confidence on how to do it hands on”*.

Drawbacks to learning when experiencing the real lab environment were reflected in the following student comment:

*“Too hard to follow and too crowded to see and have a chance to have a go in the short two hour lab”.*

### Learning of research technical skills by students

Student feedback indicated that engagement with the vLAB allowed students to make mistakes in the learning process of preparing gels, with some students commenting on how the vLAB could assist in remembering techniques for the long term.

*“I didn't have to be afraid to mess up (in) preparing the gel”; and “More practical hands on learning to help incorporate the techniques more effectively into long term memory”.*

*With regard to the real lab, typical student comments included:*

*“Allows some experience but not the full one”; and “didn't see the full picture of what technical skills are involved”.*

## Discussion

Studies regarding the effectiveness of eLearning versus face-to-face teaching have been conducted in a wide variety of contexts [6]. Reasons for improved learning outcomes include guided instructional formats with customised feedback, increased exposure to threshold concepts [8], increased student interactivity, increased student opportunities for practice, customised remediation and revision, as well as the potential to reduce redundancy and split attention both of which can lead to cognitive overload [22]. Use of the AeLP has been shown to lead to more effective student learning in many contexts already [9, 15, 23, 24]. To our knowledge, this is the first study of an eLearning intervention utilising a Western Blotting virtual laboratory.

Quantitative and qualitative data obtained from questionnaires indicate that the vLAB improved student perceptions of some aspects of their learning of Western Blotting, and was at least equivalent to the real lab in all aspects evaluated. The efficacy of the vLAB might have been in part due to the provision of an individualised Western Blotting laboratory experience, in contrast with provision of a focussed, yet limited real lab environment within the same practical class [6]. The overarching conceptual framework and technical environment provided by the vLAB appeared to provide a more comprehensive learner experience, whereby students felt better prepared for the real lab experience upon completion of the vLAB.

Students rated the Western Blotting vLAB favourably for interpreting Western Blotting for diagnosis of disease, compared with the real lab in 2011. The vLAB and real lab had equivalent effects on interpreting Western Blotting data for diagnosis of disease in 2012 and 2013. It could be that the novelty of the vLAB approach was responsible for the perceived benefits in the 2011 cohort.

Diagnostic skills are key in underpinning learning of concepts related to the molecular basis of muscular dystrophy. Student feedback demonstrated that they enjoyed being able to use the vLAB to prepare for real lab ‘hands on experience’ and generally enjoyed the interactivity and immediate feedback that the vLAB provided at each step. These data indicate that the vLAB has the capacity to facilitate learning of concepts at least as well as the real lab, according to students' perceptions. This was achieved without requiring extra resourcing or a significant extra investment of student time as judged by the time taken to achieve the learning objectives within the two-hour practical class. This suggests that the design of the vLAB provided students with a time-efficient learning experience.

This vLAB allowed students to learn concepts and techniques via dovetailing ‘real or wet’ and ‘virtual’ laboratories [4, 20, 25]. It has been shown previously that Science students have a greater understanding of best practice and diagnostic outcomes when learning by doing [24, 26]. The technical aspect of the practical class on muscle diseases was therefore important for understanding protein expression status in patients with muscular dystrophy. Students appeared to have a perceived improvement in some aspects of understanding following engagement with the Western Blotting vLAB, particularly the application of knowledge to diagnose changes in protein expression that underlie muscular dystrophy. It might be that exposing students to the vLAB allowed students to focus on the acquisition of diagnostic skills, separate from learning the technical skills of how to physically use equipment in a real lab.

The high level of interactivity and feedback provided by the vLAB, in comparison with the real lab, might have contributed to improved efficacy of delivery of diagnostic content to support learning of laboratory based concepts. This effect has been demonstrated in previous successful eLearning interventions [16, 23, 27]. Multiple studies have found that targeted eLearning environments which embed technical aspects within an overarching conceptual framework, are generally more effective than generic online texts and simulations [4, 14, 19, 28]. When considering implementation of eLearning for undergraduate students, interactivity and feedback are important for both student engagement and learning impact [12, 16, 29, 30].

The present study focuses on student perceptions, rather than objective assessment of diagnostic skills. Further studies are needed to determine whether exposure to vLABs results in equivalent skill development compared to real labs.

## Conclusion

This is the first study to report use and evaluation of an interactive virtual laboratory environment using Western Blotting in Pathology. The vLAB was perceived by students to be at least as helpful for learning as the real lab. It is envisioned that the Western Blotting vLAB could be adapted for a range of learning activities across a range of learners, undergraduate and graduate, in which a deeper understanding of protein expression patterns is required. The concepts relating to the creation of vLABs are likely to be generalisable to other domains as well. We believe that this study has important implications for the design of future vLABS to support learning of laboratory technical and diagnostic skills. Further studies of vLABs are required to elucidate the impact of vLABs on students' learning.

## Electronic supplementary material

Additional file 1: **Phases for software development and management of the Western Blotting vLAB.** (DOCX 16 KB)

Additional file 2: **Western Blotting vLAB Design.** (DOCX 18 KB)

Additional file 3: **Embedding virtual laboratories in third year undergraduate science courses to enhance student learning of research techniques.** (DOCX 49 KB)
