# Supplementary material for: Evaluation of an adaptive virtual laboratory environment using Western Blotting for diagnosis of disease
Source: BMC Med Educ. 2014 Oct 20;14:222. doi: 10.1186/1472-6920-14-222 (PMC4287185; doi:10.1186/1472-6920-14-222)
Supplement: Supplementary file 1 — Additional file 1: Phases for software development and management of the Western Blotting vLAB. (DOCX 16 KB) [file 12909_2014_1051_MOESM1_ESM.docx]

**Additional file 1**: Phases for software development and management of the Western Blotting vLAB.

*Requirements and Design*: we commenced by producing a lesson storyboard and Western Blotting vLAB requirements as a series of documents.

The documents included a description of:

- Physical Laboratory Processes
- Learning Objectives
- Processes to be made ‘virtual’ vs. demonstrated (video)
- Common student misconceptions
- Student and vLAB interactions, i.e. use cases
- vLAB lesson context, i.e. the exact nature of the vLAB-lesson integration
- vLAB scenes corresponding to distinct virtualised procedures, e.g. gel casting, sample loading and diagnostics.
- vLAB components corresponding to real-life apparatus
- vLAB lesson interface to enable tracking of student interaction with the vLAB in real time.

The documents were supplemented by reference images and videos obtained in the real laboratory of academic staff performing various parts of the experiment. The reference images and videos were utilised by a graphic designer to produce visual assets for the construction of the Western Blotting vLAB.

*Construction*: Western Blotting vLAB construction began once all requirements were clarified and visual assets produced. The production tools were Adobe Flash Professional, Adobe Flash Builder and Eclipse Integrated Development Environment (IDE). The adaptive lesson was compiled using the AeLP Author component in conjunction with vLAB construction. The lesson was consistent with the storyboard document produced during the *Requirements & Design* phase.

*User Testing and Acceptance*: we tested vLAB components throughout the development process to ensure usability and authenticity. Once all usability issues were resolved, the vLAB was accepted as ready to deploy.

*Deployment*: the Western Blotting vLAB practical lesson was deployed as part of the Musculoskeletal Diseases course in three consecutive years (2011 – n = 80; 2012 – n = 73; 2013 – n=59). All vLAB deployments took place in computer laboratory facilities during the Muscle Diseases practical class.
