# Supplementary material for: Evaluation of an adaptive virtual laboratory environment using Western Blotting for diagnosis of disease
Source: BMC Med Educ. 2014 Oct 20;14:222. doi: 10.1186/1472-6920-14-222 (PMC4287185; doi:10.1186/1472-6920-14-222)
Supplement: Supplementary file 2 — Additional file 2: Western Blotting vLAB Design. (DOCX 18 KB) [file 12909_2014_1051_MOESM2_ESM.docx]

**Additional file 2: Western Blotting vLAB Design**

The most important factors in the design of the vLAB for enhancement of learning were:

- *Authenticity*: a laboratory environment emulating the real-world or ‘wet-lab’ environment and infrastructure with particular relevance to the type of equipment available and its use.
- *Interactivity*: responded to user input, with flexibility for different learner needs which relied on differing levels of ability.
- *Feedback*: facilitates remediation of misconceptions; and
- *Integration*: provision of a conceptual framework taking into account laboratory learning objectives, which were aligned with lesson and course objectives. Theoretical concepts of the molecular basis of disease and diagnosis were facilitated by understanding laboratory techniques used to generate particular disease findings that relate to a clinical diagnostic context.

***Technical Considerations***

The system selected for the deployment of the vLAB-based lesson was the Adaptive eLearning Platform (AeLP). This is a web-based set of tools that enable educators to create, publish and analyse adaptive eLearning activities [[13-15](#_ENREF_13)].

The vLAB was deployed using web browsers with the Adobe Flash plug-in enabled.

The AeLP was selected for the following reasons:

*Accessibility*: the AeLP runs as a Software as a Service (SaaS). Therefore, it is accessible via the web without specialised software.

*Usability*: the AeLP offers a set of tools to enable the creation, publishing and analysis of rich, interactive and adaptive content. The platform includes a What-You-See-Is-What-You-Get (WYSIWYG) authoring component, which was used to design the adaptive vLAB in an intuitive manner.

*Flexibility*: the AeLP can facilitate various deployment types such as preparation for class, in-class or for homework, private or public and formative or summative assessment.

*Adaptivity*: the AeLP offers various levels of adaptivity to allow for a dynamic and personalised student learning experience whereby the feedback and the sequence of tasks and associated questions can be designed to adapt to students’ specific misconceptions. Once deployed, the lesson can be further adapted in response to issues identified during use or for deployment in a different course or institution.

*System Integration*: AeLP activities can be integrated with commonly used learning management systems (LMS) using the Learning Tools Interoperability (LTI) protocol. A Single-Sign-On (SSO) and reporting of grades (if desired) is enabled via this integration.
