# Supplementary material for: Evaluation of an adaptive virtual laboratory environment using Western Blotting for diagnosis of disease
Source: BMC Med Educ. 2014 Oct 20;14:222. doi: 10.1186/1472-6920-14-222 (PMC4287185; doi:10.1186/1472-6920-14-222)
Supplement: Supplementary file 3 — Additional file 3: Embedding virtual laboratories in third year undergraduate science courses to enhance student learning of research techniques. (DOCX 49 KB) [file 12909_2014_1051_MOESM3_ESM.docx]

**Additional file 3:**

**Embedding virtual laboratories in third year undergraduate science courses to enhance student learning of research techniques**


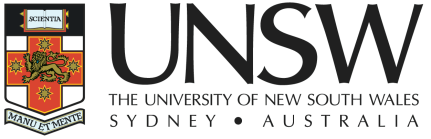


| Ethics Number | **HC13004** |
| --- | --- |

*Your assistance is sought after in providing feedback for the Muscle Diseases Practical component of PATH3207. This questionnaire is* ***anonymous.*** *Please answer each question honestly and thoughtfully. All comments and suggestions are welcome. Thank you for your time.*

1. Did the virtual laboratory improve your understanding of Western Blotting?

Yes Not Sure No

2. Did the real life laboratory improve your understanding of Western Blotting?

Yes Not Sure No

3. How confident do you feel about using the virtual laboratory ? On a scale from 1(low) to 5 (High),

Not confident confident extremely confident

1 2 3 4 5

4. How confident do you feel about using the real life laboratory ? On a scale from 1(low) to 5 (High),

Not confident confident extremely confident

1 2 3 4 5

5. **A**. Did you find the virtual laboratory (screens 1 and 2) helped you to learn technical skills about Western Blotting?

1. no, it did not

2. in most parts, no

3. in most parts, yes

4. yes

**B**. What did you find to be most valuable? _________________________________________________________________ _________________________________________________________________

6. **A**. Did you find the real life laboratory helped you to learn technical skills about Western Blotting?

1. no, it did not

2. in most parts, no

3. in most parts, yes

4. yes

**B**. What did you find to be most valuable?

Comments:______________________________________________________ _________________________________________________________________

7. **A**. Did you find the virtual laboratory (screen 3) helped you to learn diagnostic skills about using Western Blotting to understand Muscle Disease diagnosis?

1. no, it did not

2. in most parts, no

3. in most parts, yes

4. yes

**B**. What did you find to be most valuable? _________________________________________________________________ _________________________________________________________________

8. **A**. Did you find the real life laboratory helped you to learn diagnostic skills about using Western Blotting to understand Muscle Disease diagnosis?

1. no, it did not

2. in most parts, no

3. in most parts, yes

4. yes

**B**. What did you find to be most valuable?

Comments:______________________________________________________

_________________________________________________________________

9. **A**. Overall, did you find you learnt more valuable skills from the real life laboratory or the virtual laboratory?

Real Lab Not sure Virtual Lab

**B**. Describe the skills you felt you acquired from the more useful laboratory.

Comments:______________________________________________________

_________________________________________________________________

10. If you completed the virtual lab first, do you think it would have been more useful to do the real life wet lab first?

Yes Not Sure No

Why?

11. If you completed the real life wet lab first, do you think it would have been more useful to do the virtual lab first?

Yes Not Sure No

Why?

12. Please add any additional comments.

Thank you for your time.
